# An Infrared Image Defect Detection Method for Steel Based on Regularized YOLO

**DOI:** 10.3390/s24051674

**Published:** 2024-03-05

**Authors:** Yongqiang Zou, Yugang Fan

**Affiliations:** Faculty of Information Engineering and Automation, Kunming University of Science and Technology, Kunming 650500, China; masjinx@stu.kust.edu.cn

**Keywords:** infrared image, defect detection, regularization, cross-entropy, YOLOv8

## Abstract

Steel surfaces often display intricate texture patterns that can resemble defects, posing a challenge in accurately identifying actual defects. Therefore, it is crucial to develop a highly robust defect detection model. This study proposes a defect detection method for steel infrared images based on a Regularized YOLO framework. Firstly, the Coordinate Attention (CA) is embedded within the C2F framework, utilizing a lightweight attention module to enhance the feature extraction capability of the backbone network. Secondly, the neck part design incorporates the Bi-directional Feature Pyramid Network (BiFPN) for weighted fusion of multi-scale feature maps. This creates a model called BiFPN-Concat, which enhances feature fusion capability. Finally, the loss function of the model is regularized to improve the generalization performance of the model. The experimental results indicate that the model has only 3.03 M parameters, yet achieves a mAP@0.5 of 80.77% on the NEU-DET dataset and 99.38% on the ECTI dataset. This represents an improvement of 2.3% and 1.6% over the baseline model, respectively. This method is well-suited for industrial detection applications involving non-destructive testing of steel using infrared imagery.

## 1. Introduction

During the process of steel production and application, the occurrence of surface defects is inevitable. These defects can have a significant impact on the performance and lifespan of the steel. Consequently, it is particularly urgent and necessary to conduct in-depth research and solve the problem of steel surface defect detection [1].

In the industrial production process, the detection of steel surface defects often relies on manual visual inspection. However, the product quality of this inspection is limited by the inspector’s experience, and while it consumes significant manpower, it also suffers from issues of missed and false detections. With the advancement of machine vision technology, image and vision processing methods have been increasingly utilized for surface defect detection. In 1983, Honeywell in the United States developed a CCD-based surface defect detection device, significantly contributing to the prominence of machine vision in this field [2]. Feature extraction is the core of the defect detection task. Guo et al. [3] proposed an edge detection algorithm combining Kirsch and Canny operators for defect feature extraction. This algorithm has been employed for detecting bubbles and pit defects on ceramic bowl surfaces. Nieniewski et al. [4] developed a rail defect feature extraction system based on morphology. Zhou Shiyang [5] proposed a method for steel plate surface defect image detection, leveraging visual saliency and sparse representation through the dual low-rank and sparse decomposition method. It is difficult for the design of traditional machine vision algorithms to meet the diverse needs of various defect detection scenarios. With the rapid development of deep learning, establishing defect detection models based on this technology has emerged as a current research hotspot.

This article proposes a Regularized YOLO defect detection model based on YOLOv8. The model integrates the position encoding attention mechanism with C2F in the backbone, creating a lightweight CA-C2F structure that effectively captures both local and global information in defect images. Moreover, BiFPN’s learnable weighted fusion is introduced to replace the original Concat structure, enhancing the model’s ability to fuse multi-scale feature information. Finally, the model’s loss function is subject to regularization constraints, and the model parameter values are compressed to enhance generalization performance. The main contributions of this study are as follows:

(1) This study employs position-encoded attention to enhance feature information, creating a lightweight attention module with C2F that improves the feature extraction capabilities of the detection model’s backbone, particularly for steel defect edges and textures.
(2) This study introduces BiFPN’s weighted fusion strategy for multi-scale feature maps, replacing the original Concat model with BiFPN-Concat, which more effectively integrates the rich semantic information of high-level feature maps and the detailed textures of low-level feature maps. This enhances the model’s ability to extract multi-dimensional feature information.
(3) This study applies regularization constraints to the loss function and compresses model parameter values, aiding the model in learning a more concise representation and enhancing its generalization performance.
(4) This study presents an eddy current infrared image dataset of steel defects and proposes a steel infrared image defect detection model based on Regularized YOLO, aiming to provide a reference for related research.

The paper is organized as follows: Section 2 introduces related target detection algorithms. Section 3 provides a detailed description of the proposed Regularized YOLO detection model. Section 4 verifies the model’s effectiveness through a series of experiments. Finally, Section 5 concludes the article.

## 2. Related Works

Infrared image defect detection is a method that uses infrared radiation emitted by objects for detection and analysis. It is especially suitable for discovering defects and anomalies in materials or structures. With the advancement of computer technology and the development of digital imaging technology, infrared imaging equipment has begun to incorporate more advanced image processing functions, greatly improving the accuracy and efficiency of defect detection. Today’s infrared thermography technology can provide high-resolution thermal maps for more precise detection and analysis of small temperature differences, which is essential for early detection of material defects. The introduction of deep learning and artificial intelligence has further enhanced the ability of infrared image defect detection, which can automatically identify complex defect types and improve detection efficiency and accuracy.

Target detection algorithms in deep learning can be broadly classified into two groups based on their structure: the two-stage algorithms, such as Faster R-CNN [6], and the one-stage algorithms, such as SSD (Single Shot Multi-Box Detector) [7] and YOLO (You Only Look Once) [8,9,10,11,12,13]. These algorithms are predominantly data-driven and are extensively applied in the field of defect detection.

(1) Two-Stage Algorithms

A two-stage target detection algorithm exhibits higher accuracy but requires a longer processing time. It initially determines the position of the object and subsequently recognizes the position region, representing a target detection algorithm based on the candidate region. Xiang et al. [14] enhanced Faster R-CNN by substituting the coarse Region of Interest Pooling (ROI Pooling) with Region of Interest Align (ROI Align), thus achieving more accurate localization of aluminum surface defects. Cha et al. [15] proposed an enhanced Faster R-CNN to detect concrete and steel defects, modifying the structure of the Region Proposal Networks (RPNs) of ZFNet.

A two-stage algorithm demonstrates superior performance in targeting large objects and complex scenes, owing to its distinct candidate region stage that determines the object’s coordinate position. However, it necessitates computation across two network stages, leading to disadvantages such as large computational volume and slower running time.

(2) One-Stage Algorithms

A one-stage target detection algorithm forgoes the separate candidate area screening stage, instead relying on direct grid regression for position coordinates and category probabilities. In small target detection, a one-stage algorithm tends to generate a high number of candidate frames, resulting in a high false detection rate and lower accuracy compared to a two-stage algorithm. Despite these drawbacks, its fast detection speed renders it suitable for real-time detection tasks in industrial production. At present, the YOLO series of algorithms is commonly used in industry to complete detection tasks.

In the actual infrared image collection process, affected by the camera and the environment, the infrared image will suffer from problems such as loss of details at the edge of the heat source and low spatial resolution. To solve the problem of accuracy loss caused by the loss of infrared image texture features, Bao et al. [16] proposed a Dual-YOLO model for infrared and visible light image fusion based on YOLOv7, which uses attention fusion and fusion shuffling design to obtain fusion information in feature extraction and reduce the impact of redundant information. Zhang et al. [17] proposed an infrared scene target detection model YOLO-infrared based on YOLOX, which uses the attention module to extract the positional relationship between distant pixels and enhance the feature extraction capability of the model. Mou et al. [18] proposed a YOLOv5 target detection algorithm based on feature resampling to reduce the feature loss that exists in the sampling process of infrared images, scaling the size of the feature map while maintaining the current amount of feature information, and achieving a mAP of 97.4% in the relevant dataset. Hao et al. [19] applied non-negative matrix factorization (NMF) to construct the feature space in the infrared domain. They calculated the cosine similarity with the visible sample space to facilitate domain transfer. Furthermore, they combined the shallow and deep features extracted using ASFF based on YOLOv5, achieving an accuracy of 98.6% in steel plate crack detection. Zhou et al. [20] proposed a wind turbine blade detection method that uses RGB and IR images for feature fusion. It can dynamically fuse RGB images and IR images, and use the complementary features of infrared and visible light domains to identify actual defects. Tao et al. [21] propose an attention multi-hierarchical feature fusion network (AMHNet) to identify defects, using a shared aggregation gate (SAG) that dynamically adjusts the input feature mapping to complete the selection of features at different levels. Zhao et al. [22] designed a dual-feature pyramid network (DFPN) to enhance the neck and generate a rich representation for the RDD-YOLO model, thereby deepening the entire network and emphasizing low-level features.

In summary, the application of deep learning algorithms in the field of infrared shows good application prospects, and this paper improves YOLOv8 and proposes a steel infrared image defect detection model based on Regularized YOLO, which has higher robustness in dealing with the task of steel infrared image defect detection compared with the baseline model.

## 3. Steel Infrared Image Defect Detection Model

**Figure 1:**
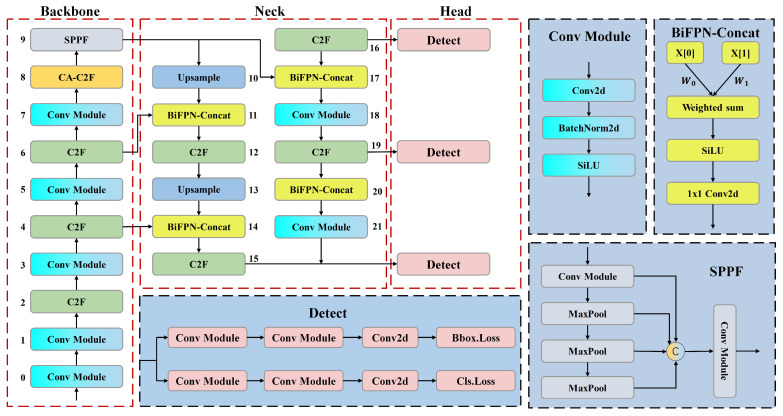
The network structure of infrared image defect detection model.

Currently, there is limited research on defect detection in infrared images. Existing models have shown limited sensitivity in detecting small defects or subtle differences, and they also lack robustness. Specifically, in the eddy current thermography dataset of steel surface defects used in this study, the class scale of defects exhibits significant variation, and the quality of the defect images is compromised by thermal diffusion, posing the challenge of blurred edge features. When employing YOLOv8 as the baseline model for defect detection, it is observed that the accuracy for certain categories is low and the model exhibits insufficient generalization performance. Consequently, this study integrates the CA (Coordinate Attention) [23] into the backbone of YOLOv8, aiming to bolster the backbone’s capture of spatial information, and thus enhance the model’s ability to characterize defective features at critical edges. Simultaneously, the fusion strategy of BiFPN [24] is employed on the neck side, replacing the standard Concat operation to amplify the feature fusion of the network for multi-scale feature maps. Furthermore, regularization constraints are introduced into the loss function to improve the model’s generalization performance in defect detection. Figure 1 shows the network structure of the proposed infrared image defect detection model.

### 3.1. Lightweight CA-C2F with Embedded Attention

Eddy current thermography utilizes the thermal effect generated by eddy currents to detect material defects. When the heat spreads from the heated area to the surroundings, the information on the edge region of the infrared defect image is easily lost, and there is the challenge of blurring in the edge features. Therefore, the goal is to compensate for the shortcomings of YOLOv8 in recognizing important information in defect images and to improve the targeting when it comes to spatial information. In this study, the CA attention mechanism is embedded in the layer 8 C2F (CSPDarknet53 to 2-Stage FPN) structure of the baseline model, so that the model obtains a lightweight attention module, which can significantly enhance the model’s ability to perceive the defect edges and improve the extraction efficiency of the texture information features.

**Figure 2:**
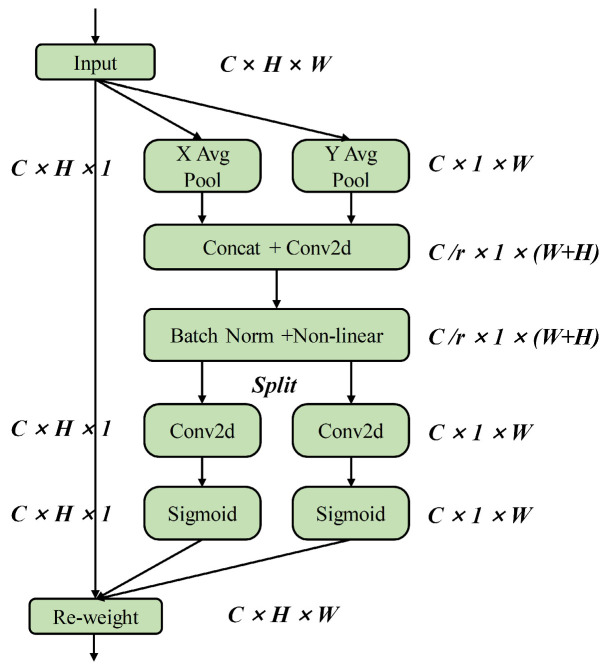
The module structure of Coordinate Attention.

The CA captures long-range spatial interactions with precise location information through the following two steps: embedding coordinate information and generating Coordinate Attention weights. As illustrated in Figure 2, the input undergoes initial average pooling, then the channels are encoded in the horizontal and vertical directions, respectively. The output z is indicative of the encoding result of the *C*-th channel with height H, and is formulated as:

(1) $$z_{c}^{h}\left(h\right)=\frac{1}{W}\sum_{0\leq i\leq W}x_{c}(h,i),$$

The output z for the *C*-th channel with width W is expressed as:

(2) $$z_{c}^{w}\left(w\right)=\frac{1}{H}\sum_{0\leq j\leq H}x_{c}(j,w),$$

Subsequently, the generated feature maps are encoded into attention weight vectors for directional awareness and position sensitivity, respectively. These vectors are then applied to the input feature maps in a complementary manner to facilitate an enhanced representation of defect features. Finally, the output $y_{c}\left(i,j\right)$ of the CA is formulated as:

(3) $$y_{c}\left(i,j\right)=x_{c}(i,j)\times g_{c}^{h}(i)\times g_{c}^{w}(j),$$

Among them, $x_{c}(i,j)$ represents the original input feature map, while $g_{c}^{h}(i)$ and $g_{c}^{w}(j)$ are the defect feature attention weight vectors obtained along the horizontal and vertical directions, respectively.

**Figure 3:**
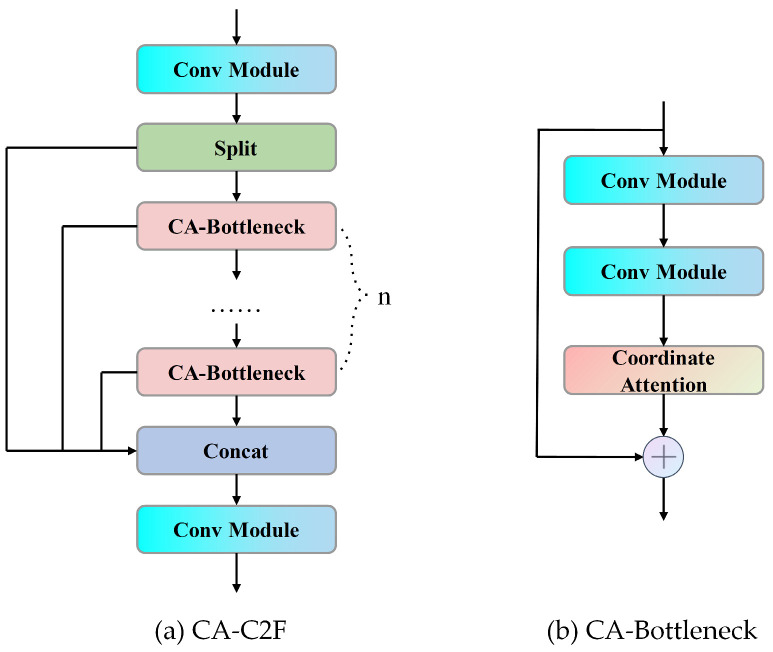
The module structure of CA-C2F.

YOLOv8’s C2F structure is characterized by two forms. In the backbone’s C2F structure, the bottleneck includes skip connections, whereas in the neck’s C2F, these are absent. The primary function of C2F lies in fusing low-level feature maps with high-level feature maps. Low-level feature maps possess more detailed information but are deficient in rich semantic and contextual information. High-level feature maps are replete with semantic and contextual information but compromise detailed information. C2F effectively integrates the advantages of both low-level and high-level feature maps. Importantly, the 8th layer of C2F acts as the input for the spatial pyramid pooling layer. As shown in Figure 3, this study embeds the CA into the bottleneck of the 8th layer C2F, aiming to improve C2F’s edge perception of low-level feature maps and the texture information feature extraction capabilities of high-level feature maps. This approach not only highlights significant defect information but also bolsters the acquisition of feature map details and semantic information, thereby significantly enhancing the accuracy of target detection.

### 3.2. Introducing BiFPN-Concat with Fusion Strategy

In order to cope with the challenges in steel surface defect detection, especially the diversity of these defects in terms of scale variation, the contribution of high-level feature maps and low-level feature maps to the model should be different, but the original Concat structure does not differentiate between the features. This paper introduces the weighted fusion strategy of BiFPN to create BiFPN-Concat. Different weight values are set to distinguish the fusion contribution of the feature maps, enhancing the fusion ability of the neck end to the defective features of different scales when splicing the feature information. This improves the accuracy and efficiency of the detection.

Traditional feature fusion approaches treat all input features equally, overlooking the contribution of feature images of different sizes to the fused output. BiFPN’s fusion strategy assigns an additional weight to each input, enabling the network to ascertain the importance of each input feature during training and to continuously adjust the fusion weights. The formula for adjusting the fusion weights method is as follows:

(4) $$O=\sum_{i}\frac{w_{i}\cdot I_{i}}{\in +\sum_{j}w_{j}},$$

where $w_{i}$ represents a learnable weight, with ReLU applied to ensure $w_{i}$ remains non-negative $\left(w_{i}\geq 0\right)$, $I_{i}$ denotes the feature of the *i*-th layer, and a small constant (∈ = 0.0001) is introduced to avoid numerical instability.

This study introduces the weighted fusion strategy of BiFPN at the neck side of the model, replacing the original Concat module with BiFPN-Concat. This new module performs a weighted fusion of two input feature maps and adjusts channel numbers via a 1 × 1 convolutional layer, employing the Swish activation function. Compared with the previous Concat module, BiFPN-Concat offers a more advanced feature fusion and transformation mechanism, making it better suited for infrared image defect detection applications that necessitate precise control over the feature fusion process.

### 3.3. Cross-Entropy Loss Function with Regularization Constraints

Conventional algorithm improvements inevitably increase model complexity and often ignore the generalization performance requirements of the model. In the current deep learning model design, how to balance the complexity and generalization ability of the model is an important challenge. Although complex model structures often achieve high accuracy on specific training datasets, their excessive complexity can also result in overfitting. To ameliorate this problem and improve the generalization performance of the model on various types of defect detection, an effective strategy is to introduce regularization constraints in the loss function to control the complexity of the model and improve the generalization ability. In this study, a key adjustment is made to the loss function of the benchmark model: regularization constraints on the structural parameters of the model are added to the cross-entropy loss.

YOLOv8’s loss function comprises a weighted sum of Classification Loss and Bounding Box Loss, with the specific formula being:

(5) $${Loss}_{total}=\omega_{1}BCE+\omega_{2}CIOU+\omega_{3}DFL,$$

where the BCE (Binary Cross-Entropy) is used for classification loss, while border regression loss encompasses CIOU (Complete-IOU) and DFL (Distribution Focal Loss).

BCE is widely used in machine learning and deep learning, especially when dealing with binary classification problems, which measures the difference between the actual labels and the model predictions. In this study, we consider adding regularization constraints based on model structural parameters to the binary cross-entropy loss function, constituting the Regularized Binary Cross-Entropy (RBCE) loss to improve the generalization performance. The RBCE loss function is defined as follows:

(6) $${RBCE}_{loss}=-\frac{1}{N}\sum_{i=1}^{N}\left[y_{i}\cdot \log\left(p_{i}\right)+\left(1-y_{i}\right)\cdot \log\left(1-p_{i}\right)\right]+\frac{\lambda }{2}{\left\|w_{i}\right\|}_{2},$$

The first part of this formula represents the standard binary cross-entropy, followed by the regularization constraint term. $w_{i}$ denotes the model’s weight parameter, trained using YOLO’s pre-training weights in this study, $w_{i}$ is the weight parameter of the model structure therein, and λ is a hyper-parameter that controls the degree of regularization.

By incorporating penalty terms, the values of the weight parameters are compressed, potentially reducing them to values near zero, thereby decreasing the model’s spatial complexity. This avoids over-reliance of the model on specific features in the training data and results in a more concise and universally applicable feature representation during the learning process. This method not only aims to reduce prediction errors in the training data but also places greater emphasis on controlling the model’s spatial complexity. It is hoped that the compressed parameters will eliminate unnecessary focus by the model, thereby optimizing its overall performance.

## 4. Datasets and Experiments

### 4.1. Related Experimental Datasets

This study used the eddy current thermal imaging (ECTI) dataset of steel surface defects and the public dataset NEU-DET to train and verify the model. Eddy current thermal imaging, a non-destructive testing method, effectively detects defects like cracks, voids, and interlayers within materials or on less visible surfaces. In the infrared scenario, defect images are influenced by temperature variations, often leading to blurred defect edges and, consequently, issues with inaccurate feature extraction and recognition.

**Figure 4:**
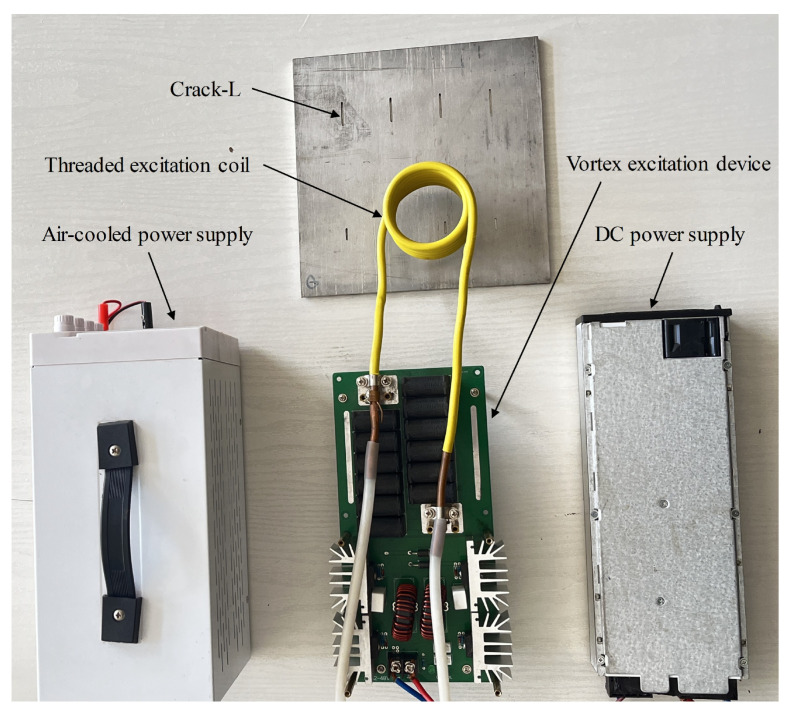
Pulse eddy current testing device.

The experimental platform of the ECTI dataset is shown in Figure 4. The maximum voltage generated by the eddy current excitation device in the experimental platform was 50 V, the maximum current was 60 A, and the excitation frequency was 60–70 kHz. The spiral excitation coil, constructed by winding six turns of highly conductive hollow copper tubes (0.8 cm in diameter), was positioned parallel and approximately 5 cm above the test piece. An infrared thermal imaging camera was used to capture infrared images and create an ECTI dataset, which enables qualitative and quantitative detection of defects by analyzing the difference in eddy current density distribution presented on the defective and intact steel surfaces.

**Figure 5:**
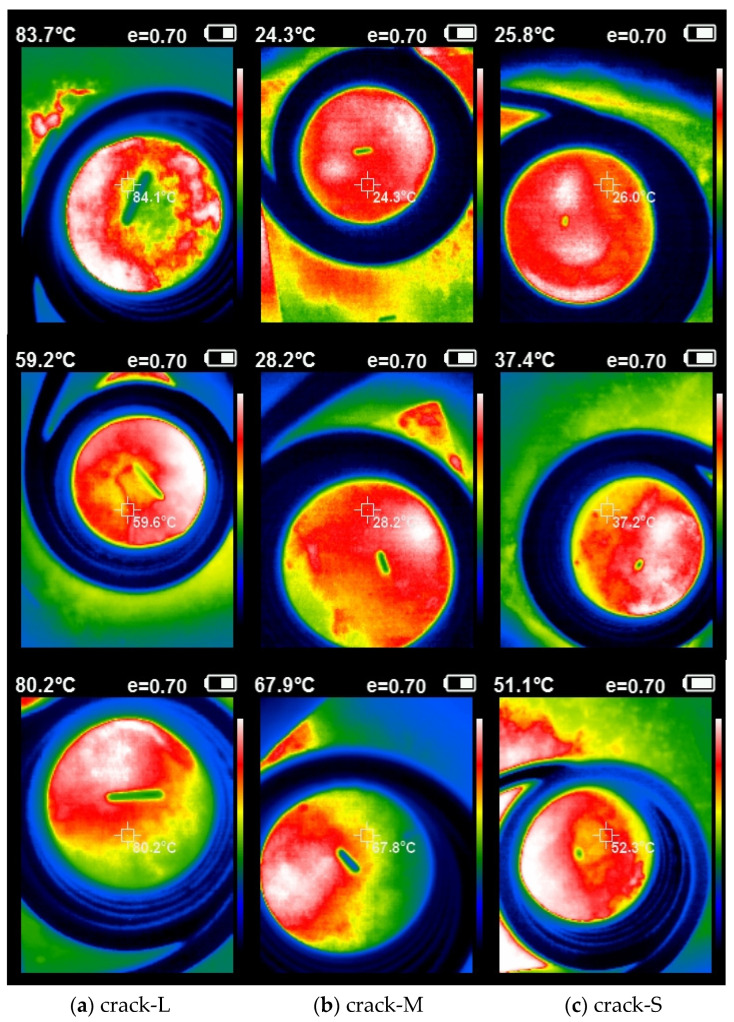
Some steel defect samples in ECTI.

The NEU-DET dataset contains 1800 images of six typical visible-light metal surface defects, including Crazing (Cr), Inclusion (In), Patches (Pa), Pitted Surface (Ps), Rolled-in Scale (RS), and Scratches (Sc). The ECTI dataset comprises 1280 images featuring three distinct sizes of eddy current thermal imaging steel crack defects. Table 1 displays the crack parameters for each of the three sizes. The dataset focuses on detecting steel surface defects with varying crack lengths but consistent width and depth. Figure 5 displays some of the crack defect samples included in the dataset.

### 4.2. Evaluation Indicators and Experimental Settings

This experiment uses the metrics of mean Average Precision (mAP0.5), Precision (P), Recall (R), F1 Score, Floating Point Operations (FLOPs), and Parameter (Params) to evaluate the model performance.

Precision indicates the probability of a sample that is actually positive among all samples that are predicted to be positive. The recall represents the probability of a sample that is actually positive among samples that are predicted to be positive. The F1 score is a balance point between precision and recall. Their formulas are as follows:

(7) $$P=\frac{TP}{TP+FP},$$

(8) $$R=\frac{TP}{TP+FN},$$

(9) $$F1=2\cdot \frac{Precision\cdot Recall}{Precision+Recall},$$

where True Positive (TP) represents the number of defects correctly identified by the model, False Positive (FP) refers to the instances of non-defective surfaces incorrectly classified as defective, and False Negative (FN) signifies the instances of defects that the model failed to detect.

The experimental environment of this study was a Windows 11 operating system with 90 GB of RAM, CPU was AMD EPYC 7T83, and GPU was NVIDIA GeForce RTX4090 (24 GB). The deep learning framework was Pytorch2.0.0, python3.8 (ubuntu20.04), Pycharm2023, and Cuda version 11.8. The training set to validation set ratio was 8:2, and the number of training rounds was set to 300 with a batch size of 32.

### 4.3. Regularisation Constraint Experiments

**Figure 6:**
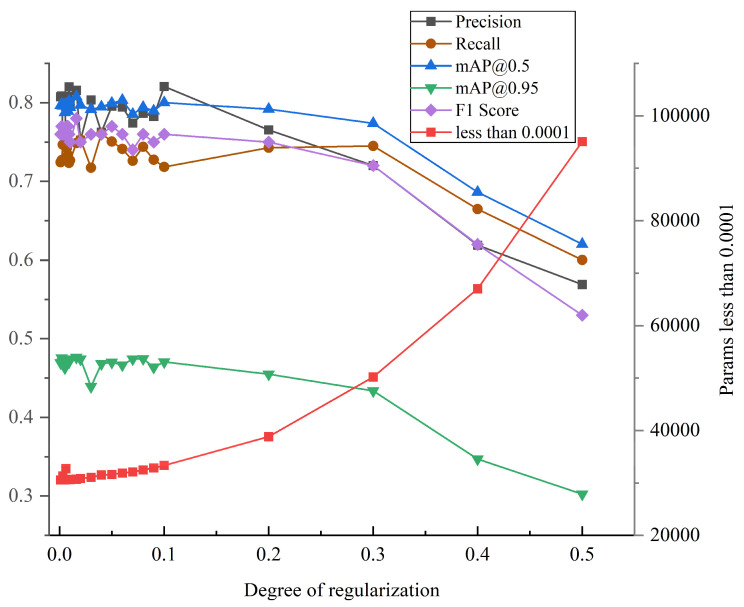
Model performance at different degrees of regularization.

This section examines the effect of introducing regularization constraints into the loss function upon the model. The effect of adding the regularization term to the model is verified by adjusting the degree of regularization and recording the trend of other parameters. The experimental data in this section are based on the publicly available dataset NEU-DET. As illustrated in Figure 6, when the degree of regularization is initially low, the various metrics of the model, such as the precision, the recall, and the F1 score, exhibit minor fluctuations on the basis of the original model. However, as the degree of regularization gradually increases, the model’s performance starts to be influenced by parameter compression, resulting in a gradual decline in the indicators. This indicates that the introduced regularization terms limit the complexity of the model to a certain extent and prevent overfitting, but excessive regularization results in a decline in model performance on the training data.

The model parameters were monitored by setting a threshold of 0.0001. As shown in Figure 6, the number of parameters with values less than 0.0001 tended to increase with higher degrees of regularization. At the initial stage, when the degree of regularization is low, the model parameters tend to have larger values overall, which can lead to overfitting the training data. As the degree of regularization increases, the model incurs stronger penalties and the parameters become more compressed, tending towards smaller values. This enhances the model’s robustness against input data and reduces its sensitivity to noise and outliers, thus improving the model’s generalization performance. This implies that regularized binary cross-entropy not only suppresses model complexity, but also encourages the model to learn a more concise representation. Given the correlation between model performance metrics and the acquisition of a more succinct representation, striking a balance between the model’s capacity to fit and generalize is achievable by implementing a lower degree of regularization.

### 4.4. Ablation Experiments

In order to verify the effectiveness of each improvement in practical applications, improvement effectiveness experiments were conducted on two datasets, NEU-DET and ECTI, respectively, to observe the trend of the model’s performance indicators.

#### 4.4.1. Ablation Experiments on ECTI

Table 2 presents the results of the progressive ablation experiments on ECTI. It is evident that the C2F-embedded CA based on the baseline has almost no increase in computation and parameters, while improving all performance indicators, particularly mAP@0.5 and mAP@0.95. This indicates a significant improvement in model accuracy at high IOU thresholds. This improvement demonstrates that embedding the CA enhances the model’s accuracy in locating objects within the backbone. However, a decrease in the recall was observed. Preliminary analysis suggests that the infrared image is affected by the ‘blurring effect’ of heat transfer, resulting in edge blurring problems, and the lightweight attention module CA-C2F does not fully account for the defective characteristics of blurred edges, resulting in missed positive samples. This performance degradation is related to the scarcity and uncertainty of fuzzy defect features in the dataset. In future research, a pre-processing step of edge enhancement for infrared images should be considered.

Upon further substituting the Concat module on the neck side of the model with BiFPN-Concat, as evidenced by the ECTI dataset performance, the introduction of BiFPN-Concat improved various model performance indicators, with the recall and F1 Score reaching the optimal 98.38% and 0.98. BiFPN-Concat’s weighted multi-scale fusion of feature maps not only combines contextual information and detailed texture information, but also enhances the model’s ability to detect inconspicuous blur edge features at the original feature level. The enhancements in mAP@0.5 and mAP@0.95 reflect BiFPN-Concat’s enhanced model performance across different IOU thresholds. It is also observed that the addition of BiFPN-Concat resulted in an increase in FLOPs from 8.2 G to 8.3 G and Params from 3.01 M to 3.03 M. Although this increase in computational and spatial complexity is marginal, to mitigate the risk of overfitting arising from model complexity, the further incorporation of regularization constraints is considered to enhance generalization performance.

The purpose of introducing the regularization term is to optimize the model’s performance and generalization ability while maintaining computational and low parameter complexity. Experimental results demonstrate that, after adding regularization constraints, the model’s accuracy reached 97.23%, with mAP@0.5 and mAP@0.95 achieving an optimal performance of 99.38% and 79.41%, respectively. These improvements indicate that the regularization term achieves a balance between improving model prediction accuracy and overall performance in industrial defect detection scenarios with limited computational resources when high performance is required. The model weight parameters are gradually compressed to near-zero values by adding regularization terms to the binary cross-entropy loss function. Although these near-zero parameters are still used in the computation, their main purpose is to reduce the impact of unimportant model parameters on overall model performance. The regularization term improves the model’s generalization ability and reduces the risk of overfitting, but it also results in the model being less sensitive to certain positive examples.

#### 4.4.2. Ablation Experiments on NEU-DET

Table 3 presents the results of the progressive ablation experiments on ECTI. The first improvement to the model showed a consistent trend of performance enhancement in the ECTI dataset, where it continued to perform excellently at higher IOU thresholds. Specifically, the mAP@0.5 increased by 1.33%, mAP@0.95 increased by 0.73%, and accuracy improved by 0.78%. This indicates that the CA-C2F lightweight module constructed in this paper indeed has a positive effect on enhancing the model’s performance.

The second improvement of the model lies in the weighted fusion of features at different levels by BiFPN-Concat. According to the experimental results, the accuracy increased from 78.26% to 81.27%, indicating that the improved model is more accurate in predicting positive classes. The differentiation in the contribution of multi-scale features to fusion indeed can enhance the model’s performance. Moreover, it achieved the highest value in the more stringent metric of mAP@0.95 in the table, reaching 47.82%. Although the F1 score had a slight improvement over YOLOv8n, this increase was not as significant as other metrics, which might be due to a slight decrease in the recall rate, dropping by 0.5% compared to before.

**Figure 7:**
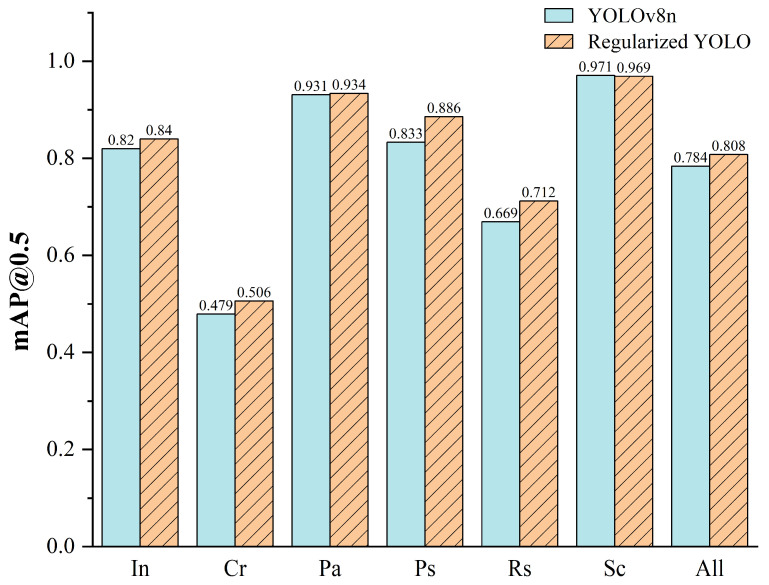
The mAP@0.5 of Regularized YOLO compared with YOLOv8n.

The third improvement of the model involves the introduction of a regularization term in the loss function. According to the experimental results, the model showed improvements across multiple key performance indicators, especially in Precision, Recall, mAP@0.5, and F1 metrics. Moreover, the addition of the regularization term seems to offset the decrease in recall rate caused by BiFPN to some extent, while maintaining its advantages in accuracy and mAP. Although mAP@0.95 was slightly decreased by 0.2%, this is usually desirable in practical applications because regularization makes the model more prone to generalization rather than optimization for specific scenarios. Figure 7 shows the mAP@0.5 of the baseline model and the proposed Regularized YOLO across different defect categories in the dataset. The mAP values for Cr and Rs defect categories show significant improvements, and except for a 0.5% decrease in Pa, the other five categories have improved, enhancing the model’s generalization performance across all categories of defects.

In summary, the three improvements discussed in this study have led to enhancements across various performance metrics. The final Regularized YOLO model demonstrated in this research shows a 4.1% increase in accuracy and a 2.3% increase in mAP@0.5 in the NEU-DET dataset compared to the baseline model. In the ECTI dataset, it achieved an accuracy of 97.2% and a 1.6% increase in mAP@0.5. These results meet the performance requirements for detecting defects in steel, confirming the advanced nature of the proposed Regularized YOLO model.

### 4.5. Comparison with the Other Model

**Figure 8:**
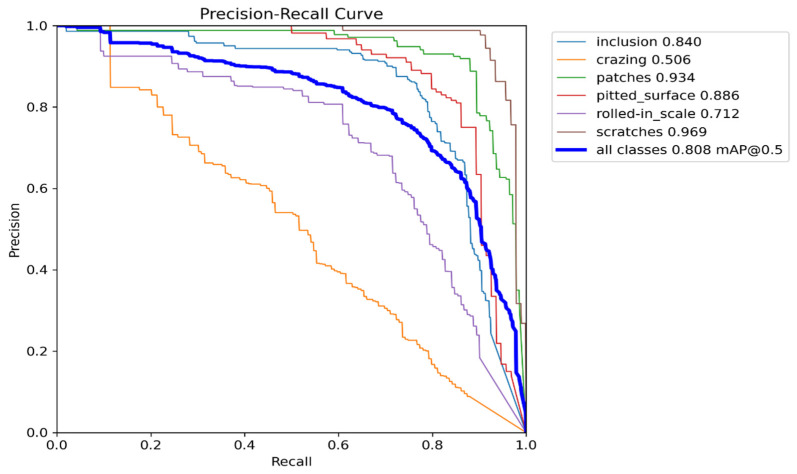
The PR curve of Regularized YOLO on NEU-DET.

To verify the performance of the model among similar algorithms, this section shows the results of comparative experiments with current mainstream detection algorithms. As shown in Table 4, the mAP@0.5 of Regularized YOLO proposed in this study reached 80.77% in the NEU-DET dataset. Compared with the one-stage algorithm SSD (VGG16), the two-stage algorithm Faster R-CNN (Resnet50), YOLOv5n, YOLOv7tiny (1 × 1), YOLOv7tiny (0.33 × 0.25), it increased by 6.8%, 4.2%, 2.6%, 10.7%, 3.9%, and 2.3%, respectively. The Precision–Recall (PR) curve of the Regularized YOLO in the NEU-DET dataset is illustrated in Figure 8.

**Figure 9:**
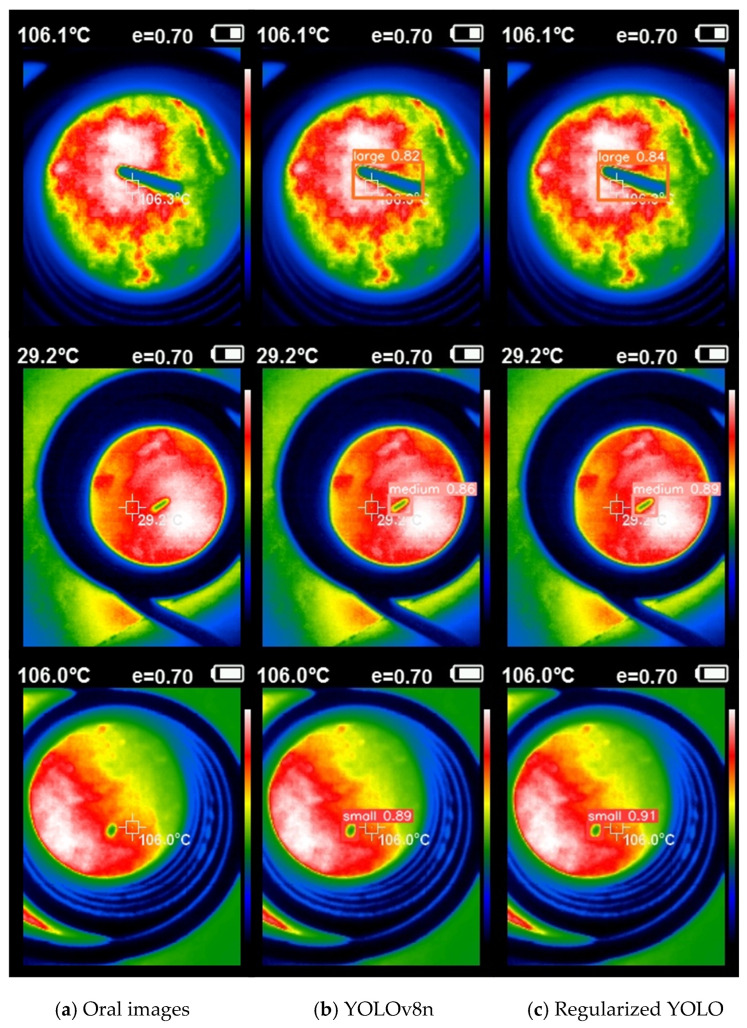
Detection plot of partial ECTI.

As indicated in Table 5, the mAP@0.5 of Regularized YOLO introduced in this study achieved the highest value of 99.38% in the ECTI dataset. In comparison with YOLOv5n, YOLOv7tiny, and others, Regularized YOLO demonstrated improvements of 1.65%, 0.68%, 0.49%, and 1.53%, respectively. Additionally, the mAP@0.5 for each defect category reached its peak. Figure 9 shows the detection results of the baseline model and Regularized YOLO on a subset of the ECTI dataset.

In the task of detecting steel surface defects in infrared domain scenarios, Regularized YOLO outperforms other models in terms of comprehensive performance. It combines the advantages of a low number of parameters and high performance, achieving a good balance between precision and recall, with higher computational efficiency. Therefore, it is fully applicable to steel surface defect detection.

## 5. Conclusions

Although conventional cameras can detect surface defects in some scenarios, eddy current thermal imaging technology is much more sensitive to changes in material temperature. This technology can detect even minute temperature differences, revealing deeper subsurface defects. This high sensitivity characteristic displayed by infrared images makes it more suitable for identifying early-stage defects in steel and preventing potential faults.

This article introduces a Regularized YOLO defect detection model for steel infrared images. It integrates the CA into the C2F module of the backbone, creating a lightweight CA-C2F, and replaces the neck’s Concat with BiFPN-Concat, thus enhancing the network. Finally, regularization constraints are infused into the model’s loss function, resulting in an optimized YOLO defect detection model. The results show that:

(1) The lightweight CA-C2F model focuses on the flow of defect features, aiding the backbone in extracting more nuanced features.
(2) The BiFPN-Concat assigns extra weights to inputs, improving the fusion of multi-scale feature maps with diverse informational focus. This strengthens the link between contextual and detailed information, boosting feature fusion capabilities.
(3) The addition of regularization constraints to the cross-entropy loss simplifies the model structure and enhances the generalization performance of the steel surface defect detection model.

In the ablation experiments, the proposed Regularized YOLO model, compared to the baseline model, achieved an improvement in accuracy with a relatively minor increase in the number of model parameters. Specifically, the parameter count increased from 3.01 M to 3.03 M, while the mAP@0.5 improved from 78.41% to 80.77% in the NEU-DET dataset and increased from 97.85% to 99.38% in the ECTI dataset, meeting the accuracy and deployment requirements for steel surface defect detection.

Although the Regularized YOLO model has achieved significant improvements for Cr and Rs types of defects, these improvements are relative, and there remains considerable room for enhancing the model’s performance for these two types of defects. Additionally, the challenge of background interference persists in the task of detecting small targets in the infrared domain.

## Figures and Tables

**Table 1:** The crack parameters on ECTI.

Type of Crack	Length (mm)	Width (mm)	Depth (mm)
Large	20	1.5	1, 2, 3, 4
Medium	8 and 10	1.5	1, 2, 3, 4
Short	4	1.5	1, 2, 3, 4

**Table 2:** The ablation experiments on ECTI.

Model	P	R	mAP@0.5	mAP@0.95	F1	FLOPs	Params
v8n	96.38	97.59	97.85	77.93	0.97	**8.2 G**	**3.01 M**
v8n+ca-c2f	96.66	96.79	98.82	78.27	0.97	**8.2 G**	**3.01 M**
v8n+ca-c2f+bifpn	96.77	**98.38**	99.32	78.81	**0.98**	8.3 G	3.03 M
v8n+ca-c2f+bifpn+l2	**97.23**	97.46	**99.38**	**79.41**	0.97	8.3 G	3.03 M

**Table 3:** The ablation experiments on NEU-DET.

Model	P	R	mAP@0.5	mAP@0.95	F1	FLOPs	Params
v8n	77.48	73.10	78.41	46.66	0.75	**8.2 G**	**3.01 M**
v8n+ca-c2f	78.26	73.72	79.74	47.39	0.76	**8.2 G**	**3.01 M**
v8n+ca-c2f+bifpn	81.27	73.22	80.73	**47.82**	0.76	8.3 G	3.03 M
v8n+ca-c2f+bifpn+l2	**81.57**	**74.87**	**80.77**	47.62	**0.78**	8.3 G	3.03 M

**Table 4:** Comparisons with other methods on NEU-DET.

Model	mAP@0.5	In	Cr	Pa	Ps	Rs	Sc	mAP@0.95	FLOPs	Params
SSD [6]	73.96	83.6	46.4	92.4	84.5	61.8	74.8	--	61.25 G	24.28 M
Faster R-CNN [7]	76.57	81.3	47.4	92.5	79.0	63.3	95.7	--	369.84 G	136.79 M
YOLOV5n	78.09	83.2	42.3	92.5	87.1	69.1	94.6	45.00	4.2 G	1.77 M
V7tiny (1 × 1) [13]	76.80	83.5	38.1	92.0	85.1	67.7	94.4	43.30	13.2 G	6.02 M
V7tiny (0.33 × 0.25) [13]	70.06	76.7	30.8	87.6	87.3	50.4	87.5	34.31	**3.4 G**	**1.51 M**
YOLOV8n	78.41	82.0	47.9	93.1	83.3	66.9	**97.1**	46.66	8.2 G	3.01 M
Regularized YOLO	**80.77**	**84.0**	**50.6**	**93.4**	**88.6**	**71.2**	96.9	**47.62**	8.3 G	3.03 M

**Table 5:** Comparisons with other methods on ECTI.

Model	mAP@0.5	L	M	S	mAP@0.95	FLOPs	Params
YOLOv5n	97.73	97.8	95.9	**99.5**	75.99	4.2 G	1.77 M
V7tiny (1 × 1) [13]	98.70	98.8	97.9	**99.5**	70.65	13.2 G	6.02 M
V7tiny (0.33 × 0.25) [13]	98.89	98.9	98.4	**99.5**	71.47	**3.4 G**	**1.51 M**
YOLOv8n	97.85	98.4	95.7	**99.5**	77.93	8.2 G	3.01 M
Regularized YOLO	**99.38**	**99.5**	**99.1**	**99.5**	**79.41**	8.3 G	3.03 M

## Data Availability

The data used to support the findings of this study are available from the corresponding author upon request.
